# A Commentary on: “Neural overlap in processing music and speech”

**DOI:** 10.3389/fnhum.2015.00330

**Published:** 2015-06-03

**Authors:** Richard Kunert, L. Robert Slevc

**Affiliations:** ^1^Neurobiology of Language, Max Planck Institute for PsycholinguisticsNijmegen, Netherlands; ^2^Neurobiology of Language, Donders Institute for Brain, Cognition and Behaviour, Radboud University NijmegenNijmegen, Netherlands; ^3^Language and Music Cognition Lab, Department of Psychology, University of MarylandCollege Park, MD, USA

**Keywords:** neural overlap, music, harmony, speech, language

## Evidence for neural overlap in processing music and speech?

There is growing interest in whether the brain networks responsive to music and language are separate after basic sensory processing or whether they share neural resources. Peretz et al.'s (2015) review on the available brain imaging evidence is a good moment to reflect on the field. We agree that “the question of overlap between music and speech processing must still be considered as an open question.” (p. 16) However, even though their review was not intended to be exhaustive, Peretz et al. (2015) have arguably focused too narrowly on neuroimaging results to give a fair assessment of current knowledge about music-language relationships.

Firstly though, it is worth re-iterating the limitations of neuroimaging studies. The fact that music experiments and language experiments reveal common brain regions (e.g., Koelsch et al., 2002; Herdener et al., 2014) is insufficient evidence for shared neural circuitry, as domain-specific neural populations might be intermingled within the same brain regions (especially given the resolution of noninvasive brain-imaging techniques). Similarly, different cognitive processes might underlie common activation sites, especially in pre-frontal areas. As just one example, attending to music over scanner noise might draw particularly strongly on prefrontal mechanisms of focused attention, compared to language perception, which might be more robust (especially in non-musicians). Therefore, Peretz et al. (2015) propose more sophisticated methods such as multivariate pattern analysis (MVPA) and adaptation paradigms. However, even these methods give equivocal interpretations: different patterns of activation in common brain areas (as revealed by MVPA) might reflect *separate* music-or-language neural populations within the same region (Rogalsky et al., 2011) or indicate the *same* neural population reacting differently to music and language (Abrams et al., 2011) possibly due to changes in functional connectivity. And while fMRI adaptation paradigms hold promise, it remains to be seen how they can be applied to this question (for two very different attempts see Steinbeis and Koelsch, 2008a; Sammler et al., 2010). Thus, the current brain imaging literature is indeed equivocal. However, looking beyond fMRI can be beneficial.

## Beyond fMRI: the interference paradigm in brain and behavior

Although Peretz et al. (2015) nicely describe the current state and limitations of functional neuroimaging evidence on music-language overlap, they ignore a large body of behavioral and electrophysiological evidence for interactive processes^1^. Much of this work relies on interference paradigms, for example, Slevc et al. (2009) asked participants to read *garden path* sentences like the following, segment by segment (while measuring reading time as a proxy for processing cost):

- After | the trial | the attorney | advised | the defendant | *was* | likely | to commit | more crimes.
- After | the trial | the attorney | advised that | the defendant | *was* | likely | to commit | more crimes.

Resolving the temporary syntactic ambiguity in (a), where “defendant” is initially misinterpreted as a direct object, causes slower reading of “*was*” than in (b), where “that” signals the correct interpretation. This syntactic garden path effect was augmented when hearing a task-irrelevant, harmonically unexpected chord during the reading of “*was*” (compared to a harmonically expected chord). This is unlikely to be due to the chord's acoustic unexpectancy, since a timbrally unexpected chord (i.e., new instrument) had no such effect. Slevc et al. (2009) interpreted their result as evidence for shared music-language resources which process structural relations. When these resources are taxed by a harmonically unexpected chord, they sub-optimally process challenging syntactic relations as in (a). See Table 1 for similar studies.

**Table 1:** **Overview of ten representative music-language interference studies**.

**References**	**Primary outcome**
**BEHAVIOR**	
Fedorenko et al., 2009	Melodic unexpectancy worsens the comprehension of syntactically complex sentences, volume unexpectancy without effect
Slevc et al., 2009	Harmonic unexpectancy slows the resolution of syntactic ambiguities but not of semantically unexpected words; timbre without effect
Hoch et al., 2011	Harmonic unexpectancy slows the word judgment time of syntactically unexpected words, but not of semantically unexpected words
Perruchet and Poulin-Charronnat, 2013	Harmonic unexpectancy slows the resolution of semantic ambiguities but not of semantically unexpected words
Fiveash and Pammer, 2014	Harmonic unexpectancy worsens sentence recall but not word list recall; timbral unexpectancy without effect
**ELECTRO ENCEPHALOGRAPHY (EEG)**	
Besson et al., 1998	Melodic unexpectancy does not affect the event-related potential (ERP) to a semantic manipulation (N400)
Koelsch et al., 2005	Harmonic unexpectancy affects the syntax-related left anterior negativity (LAN) but not the N400
Steinbeis and Koelsch, 2008b	Harmonic unexpectancy affects the LAN but not the N400; language syntactic violations affect the harmony-related early right anterior negativity (ERAN) while language semantic anomalies affect the harmony-related N500
Carrus et al., 2011	Harmonic unexpectancy affects the oscillatory response to language syntax (delta-theta bands), but not vice versa; no interaction with semantics
Carrus et al., 2013	Melodic unexpectancy affects the LAN but not the N400

*Behavioral and electrophysiological interference studies offer compelling evidence for shared musico-linguistic resources but were not discussed by Peretz et al. (2015). For illustration, we list five behavioral and five electro-encephalographical studies*.

These interference effects are compelling evidence for shared resources. While the aforementioned fMRI paradigms investigate whether shared neural circuitry is extensive enough to be visible in fMRI, studies like those in Table 1 investigate the functional relevance of shared resources (e.g., in terms of behavioral outcomes). Given the support for the latter, an important debate has centered on the functional role of shared resources, such as involvement in structural processing (Patel, 2003), general attention (e.g., Perruchet and Poulin-Charronnat, 2013), or cognitive control (Slevc and Okada, 2015). This debate would surely benefit from a variety of approaches which reveal the time-course, oscillatory, and network dynamics (e.g., via electrophysiological measures of brain activity), as well as the causal role of associated brain areas (e.g., via transcranial magnetic stimulation). Targeted fMRI studies informed by the entirety of the neural as well as the behavioral literature are needed to complement these approaches.

## Toward an inter-disciplinary science of music and language processing

Peretz et al. (2015) are certainly right when they write that “converging evidence from several methodologies is needed.” We have tried to sketch the impressive extent of the evidence that is *already* available. However, there are still open questions. For example, the interference paradigm has so far not been used with linguistic processes beyond syntax and semantics (e.g., phonology, morphology, and prosody) and musical processes beyond melody, harmony, and timbre (e.g., rhythm).

Greater insights into music and language offer great potential for example in terms of clinical applications. Specifically, syntactic processing problems found in Broca's aphasia (see Patel et al., 2008) and specific language impairment (Jentschke et al., 2008) could be helped by melody-harmony interventions given evidence for shared resources for syntax and harmony, see Table 1. Progress with such clinical applications requires us first to understand how music and language relate to each other. This understanding can only emerge when going beyond a focus on any one method and, instead, viewing the field as an inter-disciplinary challenge.

### Conflict of interest statement

The authors declare that the research was conducted in the absence of any commercial or financial relationships that could be construed as a potential conflict of interest.

## Footnotes

^1^Peretz et al. (2015) focus on music and *speech*, not *language* as we do here. However, the former has also been investigated with the interference paradigm with stimuli sung a *cappella* (Besson et al., 1998; Fedorenko et al., 2009).
